# Validation of a Spanish version of the Revised Fibromyalgia Impact Questionnaire (FIQR)

**DOI:** 10.1186/1477-7525-11-132

**Published:** 2013-08-01

**Authors:** Monika Salgueiro, Juan Miguel García-Leiva, Javier Ballesteros, Javier Hidalgo, Rocío Molina, Elena P Calandre

**Affiliations:** 1 Department of Neurosciences, University of the Basque Country UPV/EHU, Bilbao, Spain; 2 Institute of Neurosciences, University of Granada, Granada, Spain; 3 Spanish CIBER of Mental Health (CIBERSAM), Bilbao, Spain

**Keywords:** Fibromyalgia, Revised fibromyalgia impact questionnaire, Linguistic validation, Psychometric properties, Instrumental study, Fibromialgia, Cuestionario de impacto de fibromialgia revisado, Validación lingüística, Propiedades psicométricas, Estudio instrumental

## Abstract

**Background:**

The Revised version of the Fibromyalgia Impact Questionnaire (FIQR) was published in 2009. The aim of this study was to prepare a Spanish version, and to assess its psychometric properties in a sample of patients with fibromyalgia.

**Methods:**

The FIQR was translated into Spanish and administered, along with the FIQ, the Hospital Anxiety Depression Scale (HADS), the 36-Item Short-Form Health Survey (SF-36), and the Brief Pain Inventory (BPI), to 113 Spanish fibromyalgia patients. The administration of the Spanish FIQR was repeated a week later.

**Results:**

The Spanish FIQR had high internal consistency (Cronbach’s α was 0.91 and 0.95 at visits 1 and 2 respectively). The test-retest reliability was good for the FIQR total score and its function and symptoms domains (intraclass correlation coefficient (ICC > 0.70), but modest for the overall impact domain (ICC = 0.51). Statistically significant correlations (p < 0.05) were also found between the FIQR and the FIQ scores, as well as between the FIQR scores and the remaining scales’ scores.

**Conclusions:**

The Spanish version of the FIQR has a good internal consistency and our findings support its validity for assessing fibromyalgia patients. It might be a valid instrument to apply in clinical and investigational grounds.

## Abstract (es)

**Resumen:**

**Antecedentes**

El Cuestionario de Impacto de Fibromialgia (FIQ) ha sido utilizado como medida de valoración en numerosos estudios clínicos. En 2009 se publicó en inglés su versión revisada (FIQR). El presente estudio se llevó a cabo para traducir al español y validar dicha versión.

**Pacientes y Método**

La traducción fue realizada por dos de los autores. La versión traducida del FIQR se administró a 113 pacientes con fibromialgia junto con el FIQ, la Escala Hospitalaria de Ansiedad y Depresión, el Cuestionario de Salud SF-36 y la versión abreviada del Cuestionario Breve de Dolor. Una semana después se administró de nuevo el FIQR. Su consistencia interna se evaluó mediante el coeficiente *α* de Cronbach. Se calcularon los coeficientes de correlación intraclase (ICC) entre las puntaciones de la nueva versión y la anterior, así como las subescalas del Cuestionario SF-36, de la Escala Hospitalaria de Ansiedad y Depresión y del Inventario Breve de Dolor. La fiabilidad test-retest se evaluó igualmente mediante coeficientes de correlación intraclase.

El coeficiente *α* de Cronbach de la versión en estudio fue elevado (0,91 en la primera visita y de 0,95 en la segunda). La fiabilidad test-retest fue buena para la puntuación total del FIQR y para las dimensiones de función y síntomas ( ICC ≥ 0,70) pero modesta para la dimensión de impacto global (ICC = 0,51). Asimismo se encontraron coeficientes de correlación elevados y estadísticamente significativos respecto a las restantes escalas aplicadas (*p* < 0,05).

**Conclusión**

La versión española del FIQR es un instrumento válido para su uso en la evaluación e investigación clínicas.

## Background

Fibromyalgia syndrome (FMS) is a chronic musculoskeletal disorder characterized by widespread and diffuse pain, often accompanied by fatigue, sleep disturbances, and depressed mood [1]. It significantly impairs the quality of life of the patients and can be highly disabling [2]. Since neither diagnostic laboratory tests nor specific radiological findings are available for its diagnosis, the assessment of pain severity and accompanying symptoms is considered essential on this regard, as well as its impact on the functional capacity and the quality of life of FMS patients. Burckhardt et al. [3] developed and published a tool to measure the impact of FMS symptoms on daily living abilities and general health status, the Fibromyalgia Impact Questionnaire (FIQ). The authors’ purpose was to take into account not only the pain, but also symptoms such as restless sleep, fatigue, muscular stiffness, anxiety, or depression, and their impact on perceived quality of life in the previous week.

The FIQ has been translated and validated in 14 different languages, and has been used as an outcome measure in more than 300 research papers. In fact, it is considered the most sensitive method to evaluate the clinical course of the FMS as well as its response to treatment in clinical trials [4-6]. The questionnaire, however, has been criticized, mainly due to the cumbersome scoring algorithm used, and the absence of important issues such as cognitive impairment, postural balance, or environmental sensitivity [7].

In response to such criticisms, Bennett and his colleagues published, in 2009, a revised version of the questionnaire, the FIQR [8]. The improved proposed tool disposes of the Likert items and the visual analogue scales, and assesses the severity of symptoms in using discrete values shown in 11 boxes, valued from 0 to 10, with 10 being ‘worst’. Furthermore, it includes a wider range of symptoms associated with FMS, such as tenderness to touch, memory disorders, postural balance, hyperalgesia, or sensitivity to environmental factors. Some of the questions, originally intended for women living in reasonably affluent income countries in a previous FIQ, were reformulated to be suitable for both men and women of all socioeconomic levels. As in the previous version of the FIQ, all questions relate to the impact of FMS over the course of the past 7 days.

The FIQR has recently been validated and translated into Turkish [9] and to Arabian Moroccan [10]. The authors of this study undertook a translation of the FIQR (Appendix A) that was used in a previous study on the risk of suicide in FMS patients [11]. The aim of this paper was to test the reliability (internal and test-retest) and construct validity of the Spanish version of the FIQR.

## Materials and methods

### Participants

Subjects were recruited from several associations of FMS patients of different Spanish provinces. To be included in the study, patients had to fulfill the American College of Rheumatology criteria for FMS published in 1990 [1]. The only exclusion criterion was a medical, psychiatric, or cognitive disorder that impeded the patient’s ability to correctly answer the Case Report Form (CRF). In order to include a representative sample of Spanish FMS patient, age and comorbidity were not considered as exclusion criteria.

Those patients who volunteered to participate were given for completion a CRF that included sociodemographic data, the proposed Spanish version of the Revised Fibromyalgia Impact Questionnaire (FIQR), the validated Spanish versions of the original FIQ, the Hospital Anxiety and Depression Scale (HADS), the Short Form 36 Health Survey (SF-36), and the Brief Pain Inventory (BPI). All patients completed these instruments on the day of the visit, and one week later participants were reappraised by using only the FIQR.

The study protocol was approved by the Human Research Ethics Committee of the University of Granada, and all the patients signed a consent form attesting their willingness to participate in the study.

### Assessment tools

#### Fibromyalgia Impact Questionnaire (FIQ)

The FIQ [3] is a 10-item self-report questionnaire developed to measure both physical and psychological symptoms of FMS, and their interference in daily living tasks and in perceived quality of life by patients. The first item uses 4-point Likert scales for assessing patients’ ability to carry out physical activities over the course of the past 7 days. Items 2 and 3 indicate the number of days in the former week that the patients felt good, and how many days of work they missed. Finally, the remaining seven questions use a 10-centimeter visual analogue scale in order to measure patients’ disability at work, and severity of pain, fatigue, morning tiredness, muscular stiffness, anxiety, and depression. The questionnaire scoring procedure requires recoding of item 3, and standardizing raw scores for calculating the arithmetic mean value ranging from 0 (lowest impact) to 10 (maximum impact). The consensus version [12] made from the different Spanish FIQ forms was used.

### Revised Fibromyalgia Impact Questionnaire (FIQR)

The FIQR [8] is a 21-item self-administered questionnaire. All items are visual analogue scales with 11 boxes discreetly scoring from 0 to 10. From the direct answers three linked sets of domains can be calculated: (i) function, which is the sum of the first 9 items divided by 3, and can take a value between 0 and 30; (ii) overall impact, which is the sum of the items 10 and 11, and can take a value between 0 and 20; and (iii) symptoms, which is calculated by adding the raw score of the items from 12 to 21 divided by 2, and can take a value between 0 and 50. The total FIQR, whose maximal score is 100, is the sum of the three domain scores, and represents the overall impact of symptoms on quality of life.

The Spanish translation of the original questionnaire was performed and agreed by two bilingual authors of the study (EPC and JMGL) [See Additional file 1].

### Hospital Anxiety and Depression Scale (HADS)

The Hospital Anxiety and Depression Scale (HADS) [13] is commonly used to determine the levels of anxiety and depression that a patient is experiencing. This scale specifically avoids reliance on aspects of these conditions that are also common to somatic symptoms or illness, such as fatigue or insomnia, so that it is very useful in the assessment of anxiety and depression in patients with physical illness. It is a 14-item scale, seven of the items related to anxiety and seven related to depression. Each item is scored from 0 to 3 by using a Likert scale. Overall rates of either anxiety or depression can take values comprised between 0 and 21, with higher scores indicative of greater severity of symptoms. The validated Spanish version [14] was used.

### Short Form (36) Health Survey (SF-36)

The Short Form (36) Health Survey (SF-36) [15] provides a profile of health status and is often used to assess the health related quality of life [16]. The questionnaire consists of eight subscales: vitality, physical functioning, bodily pain, general health perception, physical role functioning, emotional role functioning, social role functioning, and mental health, which are the weighted sums of the questions in their section. Each scale is directly transformed into a 0–100 scale on the assumption that each question carries equal weight. Therewith, two global domains of health can be calculated, named Physical Component Summary (PCS) and Mental Component Summary (MCS). In all subscales and domains higher scores refer to better health status. The Spanish version of SF-36 adapted by Alonso and collaborators [17] was used.

### Brief Pain Inventory (BPI)

The Brief Pain Inventory (BPI) [18] is a validated, widely used, self-administered instrument developed to assess pain interference with daily functions [19]. It uses visual analogue scales ranging from 0 to 100 to measure both the intensity of the pain (sensory dimension) and its interference with the patient’s life (reactive dimension). It also queries the patient about pain relief, pain quality, and patient perception of the cause of pain. The validated Spanish version of BPI [20] was used.

### Statistics

Demographic data were analyzed using the descriptive statistics of mean, standard deviation (SD) and range. Age and duration of pain were used as continuous variables. The remaining variables were used as dichotomous ones and described using the absolute and relative frequencies.

Test-retest reliability was assessed using the intraclass correlation coefficients (ICC) between the FIQR scores of the first and the 1-week follow-up evaluations. We used a two-way random effect model to estimate the ICC and 95% confidence intervals (CI) [21]. Internal consistency was determined using Cronbach’s *α* coefficient. Construct validity was evaluated with Pearson’s *r* correlation coefficients correlating the FIQR total score with the former FIQ, the HADS, and the BPI for convergent validity, and with the SF-36 for divergent validity. Guided by previous research on FIQR validations [9,10] we expected to find internal consistency values (Cronbach’s α) ≥ 0.80, test-retest reliability values (ICC) ≥ 0.70, and convergent and divergent validity values showing at least moderate to important correlations between the FIQR and the target scales (Pearson’s *r* values in the range of 0.40 to 0.70). Given these values we estimated that sample sizes in the range of 20 to 60 subjects would be sufficient to estimate correlations between 0.40 and 0.70 assuming a 5% as significance level and 90% power.

Statistical analyses were conducted with the SPSS v17.0 (Chicago, IL, USA) and Stata v12 (College Station, TX, USA)

## Results

One hundred and fourteen patients completed the questionnaires: one of these was excluded because of missing items. Thus, the study sample consisted of 113 patients. Their sociodemographic characteristics are shown in Table 1.

**Table 1:** Sociodemographics of fibromyalgia syndrome patients (N = 113)

	**[N (%)]**
**Gender**	
- Female	109 (96.5)
- Male	4 (3.5)
**Age in years** (mean ± SD)	51.6 ± 9.6
**Marital status:**	
- Unmarried	23 (21.5)
- Married	67 (62.6)
- Divorced	13 (12.2)
- Widowed	3 (2.8)
**Education:**	
- lliterate	9 (8.0)
- Primary	51 (45.5)
- Secondary	37 (33.0)
- Tertiary	14 (12.5)
**Disease duration in years** (mean ± SD)	8.5 ±7.6

**Figure 1:**
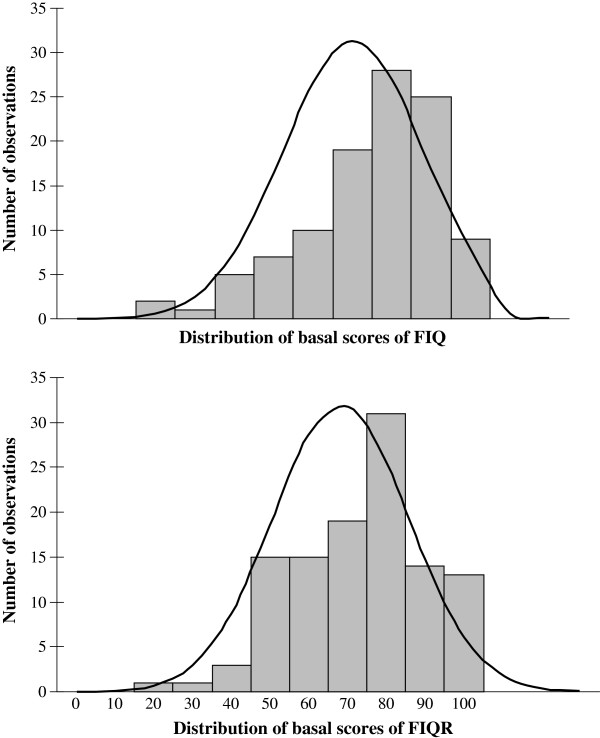
Histograms of FIQ and FIQR showing distribution of basal scores.

Table 2 shows the main descriptive statistics for the FIQR at the test and retest visits one week apart as well as the ICC to estimate the temporal stability of the measurements. The studied sample showed a FIQ total score of 70.15 (SD: 17.82). Histograms of distributions of FIQ and FIQR total scores are shown in Figure 1. Cronbach’s *α* calculation for the Spanish version of FIQR was 0.91 in the first visit, and 0.95 in the second visit.

**Table 2:** Test-retest reliability of the Revised Fibromyalgia Impact Questionnaire total, dimensions, and individual items scores

	**Visit 1 (mean ± SD)**	**Visit 2 (mean ± SD)**	**ICC (95% CI)**
**FIQR total**	68.2 ± 17.5	67.4 ± 19.9	0.82 (0.75 to 0.87)
**FIQR function**	18.9 ± 6.7	19.2 ± 6.8	0.73 (0.63 to 0.80)
Comb hair	4.48 ± 2.9	4.56 ± 3.1	0.72 (0.62 to 0.80)
Walk for 20 minutes	5.70 ± 3.4	5.75 ± 3.5	0.74 (0.64 to 0.81)
Prepare a meal	4.57 ± 3.0	5.22 ± 3.1	0.66 (0.54 to 0.75)
Clean floors	6.90 ± 2.8	7.16 ± 2.7	0.60 (0.47 to 0.71)
Carry a bag of groceries	8.23 ± 6.4	7.59 ± 2.7	0.19 (0.01 to 0.36)
Climb a flight of stairs	7.16 ± 2.6	7.25 ± 2.8	0.70 (0.60 to 0.79)
Change bed sheets	6.73 ± 2.5	6.66 ± 2.6	0.74 (0.65 to 0.82)
Sit for 45 minutes	6.74 ± 2.8	6.72 ± 2.9	0.70 (0.59 to 0.78)
Go shopping for groceries	6.36 ± 2.8	6.81 ± 2.6	0.73 (0.63 to 0.81)
**FIQR overall impact**	11.8 ± 5.6	12.0 ± 5.9	0.51 (0.36 to 0.63)
Can not achieve goals	5.91 ± 2.9	5.98 ± 3.0	0.59 (0.45 to 0.69)
Feel overwhelmed	5.88 ± 3.3	6.05 ± 3.1	0.37 (0.20 to 0.52)
**FIQR symptoms**	37.5 ± 8.7	36.1 ± 9.6	0.81 (0.73 to 0.87)
Pain rating	7.73 ± 2.2	7.65 ± 2.2	0.64 (0.52 to 0.74)
Energy rating	7.73 ± 2.3	7.72 ± 2.2	0.63 (0.50 to 0.73)
Stiffness rating	7.39 ± 2.2	6.91 ± 2.6	0.69 (0.57 to 0.78)
Sleep quality	8.62 ± 2.1	8.26 ± 2.1	0.52 (0.38 to 0.64)
Depression level	6.54 ± 3.2	6.47 ± 3.2	0.87 (0.82 to 0.91)
Memory problems	7.53 ± 2.5	7.14 ± 2.5	0.78 (0.69 to 0.84)
Anxiety level	7.18 ± 2.8	6.71 ± 2.9	0.80 (0.72 to 0.86)
Tenderness level	7.43 ± 2.7	7.28 ± 2.9	0.76 (0.68 to 0.83)
Balance problems	6.95 ± 2.5	6.49 ± 2.5	0.70 (0.59 to 0.79)
Environmental sensitivity	8.02 ± 2.2	7.67 ± 2.3	0.71 (0.61 to 0.80)

*SD* Standard Deviation, *ICC* intraclass correlation coefficient, *95% CI* 95% confidence interval, *FIQR* Fibromyalgia Impact Questionnaire Revised.

The response of the Spanish version of FIQR provided in the second visit showed a satisfactory temporal stability on the dimensions assessed by the total FIQR (ICC = 0.82, 95% CI = 0.75 to 0.87), the FIQR function (ICC = 0.73, 95% CI = 0.63 to 0.80), and the FIQR symptoms (ICC = 0.81, 95% CI = 0.73 to 0.87). On the contrary the dimension of FIQR overall impact showed a low temporal stability (ICC = 0.51, 95% CI = 0.36 to 0.63). Three items of the FIQR function presented an ICC point estimate below 0.70 (“prepare a meal”, “clean floors”, and “carry a bag of groceries”) and the item “go shopping for groceries” presented a significant time effect (p < 0.05 for the mean difference between visits). The 2 items of the FIQR overall impact presented an ICC below 0.70. Four items of the FIQR symptoms presented an ICC below 0.70 (“pain rating”, “energy rating”, “stiffness rating”, and “sleep quality”), whereas the test-retest ratings for FIQR symptoms, stiffness, and memory problems showed a significant time effect (p < 0.05).

Pearson’s *r* correlations coefficients between FIQR total, function, overall impact, and symptoms scores, and study questionnaires were calculated for testing construct validity according to convergent and divergent validity criteria (Table 3). The total scores of the FIQR and FIQ were closely correlated (*r* = 0.83, *p* < 0.01). The FIQR total, function, overall impact, and symptoms scores showed significant correlations with HADS depression and anxiety subscales, pain intensity and its interference with daily activities assessed by using BPI (*p* < 0.01 in every case). Finally, significant (*p* < 0.01) inverse correlations were also found between FIQR total and its domains’ scores, and quality of life assessed by SF-36 (Table 3).

**Table 3:** Revised Fibromyalgia Impact Questionnaire total and dimensions scores construct validity (Pearson’s correlation coefficients and 95% CI)

	**FIQR total**	**FIQR function**	**FIQR overall**	**FIQR symptoms**
**FIQ total**	0.83 (0.75 to 0.88)	0.68 (0.56 to 0.77)	0.47 (0.31 to 0.61)	0.83 (0.76 to 0.88)
**HADS total**	0.69 (0.57 to 0.77)	0.53 (0.38 to 0.66)	0.45 (0.28 to 0.58)	0.70 (0.59 to 0.79)
HADS anxiety	0.66 (0.54 to 0.75)	0.50 (0.35 to 0.63)	0.42 (0.25 to 0.56)	0.68 (0.56 to 0.77)
HADS depression	0.62 (0.49 to 0.72)	0.46 (0.30 to 0.60)	0.39 (0.22 to 0.54)	0.65 (0.53 to 0.75)
**BPI severity**	0.70 (0.57 to 0.78)	0.51 (0.36 to 0.64)	0.46 (0.31 to 0.61)	0.68 (0.56 to 0.77)
**BPI interference**	0.84 (0.78 to 0.89)	0.68 (0.57 to 0.77)	0.49 (0.33 to 0.62)	0.85 (0.79 to 0.89)
**SF-36:**				
**Physical Component Summary** (PCS)	−0.53 (−0.65 to −0.38)	−0.48 (−0.61 to −0.32)	−0.33 (−0.49 to −0.16)	−0.48 (−0.61 to −0.32)
**Mental Component Summary** (MCS)	−0.65 (−0.75 to −0.53)	−0.47 (−0.60 to −0.31)	−0.41 (−0.55 to −0.24)	−0.68 (−0.77 to −0.56)
Physical functioning (PF)	−0.68 (−0.77 to −0.57)	−0.64 (−0.74 to −0.52)	−0.35 (−0.50 to −0.17)	−0.64 (−0.74 to −0.52)
Physical role functioning (PR)	−0.43 (−0.57 to −0.26)	−0.32 (−0.47 to −0.14)	−0.32 (−0.48 to −0.14)	−0.41 (−0.55 to −0.24)
Bodily pain (BP)	−0.70 (−0.79 to −0.60)	−0.55 (−0.67 to −0.40)	−0.45 (−0.59 to −0.29)	−0.70 (−0.78 to −0.59)
General health perceptions (GH)	−0.63 (−0.73 to −0.50)	−0.46 (−0.60 to −0.30)	−0.41 (−0.56 to −0.25)	−0.64 (−0.74 to −0.51)
Vitality (VT)	−0.71 (−0.79 to −0.60)	−0.55 (−0.67 to −0.41)	−0.45 (−0.59 to −0.29)	−0.70 (−0.78 to −0.59)
Social role functioning (SF)	−0.65 (−0.74 to −0.52)	−0.50 (−0.63 to −0.35)	−0.44 (−0.57 to −0.27)	−0.63 (−0.73 to −0.50)
Emotional role functioning (RE)	−0.54 (−0.66 to −0.40)	−0.40 (−0.55 to −0.24)	−0.33 (−0.49 to −0.16)	−0.56 (−0.68 to −0.42)
Mental health (MH)	−0.69 (−0.78 to −0.58)	−0.51 (−0.64 to −0.36)	−0.41 (−0.56 to −0.25)	−0.72 (−0.80 to −0.61)

9*5% CI* 95% confidence interval, *FIQR* Fibromyalgia Impact Questionnaire Revised, *FIQ* Fibromyalgia Impact Questionnaire, *HADS* Hospital Anxiety and Depression Scale, *BPI* Brief Pain Inventory, *SF-36* Short Form Health Survey.

## Discussion

Overall, our findings suggest that the FIQR is a sufficiently reliable and valid measure of health status in Spanish patients of FMS.

The sample of patients with FMS in this study presents the expected demographic and clinical characteristics of the disorder: middle-aged women, married and with primary studies, and with several years of duration of the disease (Table 1). The duration of pain and symptoms of FMS were highly variable, probably due to the wide age range (30–87 years) of studied sample.

Participants reported similar scores of FIQR in both evaluations (68.22, SD: 17.5 and 67.42, SD: 19.89, in the first and the second visit, respectively). These values are consistent with those obtained by Srifi and collaborators [10] (65, SD: 14.5 for the first visit, and 63.2, SD: 16.6 for the second visit), and are slightly higher than those published by Ediz and his colleagues [9] (55.22, SD: 21.96 and 57.16, SD: 22.48, respectively).

The psychometric properties of the Spanish FIQR in patients with FMS were similar both to those of the original English-language validation study [8] and the two subsequent Turkish [9] and Arabic Moroccan [10] validations. Internal consistency of the Spanish FIQR was found as high as 0.91 for the first visit and 0.95 for the second visit, indicating acceptable levels of internal consistency for both assessments. This level of internal consistency of the Spanish FIQR is close to the one of Bennett et al. [8], who obtained internal consistency of 0.95, and similar than those published by both Turkish (*α* = 0.89 in the first visit, and *α* = 0.91 in the second visit) [9], and Moroccan teams (*α* = 0.91 in the first visit, and *α* = 0.94 in the second visit) [10].

The test-retest reliability of the FIQR total score evaluated with the ICC was 0.82 (Table 2), close to those obtained in the Turkish (*r* = 0.84) [9] and Moroccan (*r* = 0.84) [10] versions. No test-retest reliability of the original FIQR was performed by Bennett et al. [8].

Considering separately the three FIQR domains, symptoms showed adequate test-retest reliability (ICC = 0.81), as well as function (ICC = 0.73). However, overall impact performed somewhat lower (ICC = 0.51, 95% CI = 0.36 to 0.63), and thus point to the possibility of improvement in that domain measure. This could be due to the high variability showed by FMS patients in terms achievement goals and overwhelmed feeling and their impact in daily activities. Although the general reproducibility of the Spanish FIQR was good, the test-retest reliability of some individual items, with ICC values that varied between 0.19 and 0.87, was lower than those obtained by the other validated versions of FIQR, those had Spearman’s *rho* > 0.70 [9,10]. Of course this is not unexpected since neither Pearson’s *r* nor Spearman’s *rho* as association measures do take systematic errors for individual measurements over time into account, and thus could lead to overestimate the temporal reliability of a measurement [22]. Future uses of the Spanish FIQR, should consider the reformulation of some of the items that obtained lower reliability indexes and significant time effect over the one-week period for test-retest reliability.

Construct validity, both convergent and divergent, of Spanish translation of FIQR was evidenced by the significant correlations found between the FIQR total and domains scores with all the questionnaires used in the validation process (Table 3). As expected, and as observed in previous studies [8-10], both the total FIQR and its domains showed a good correlation with the FIQ, the HADS, the BPI and the SF-36 scores. A noticeable correlation between FIQR and previous FIQ (*r* = 0.83, *p* < 0.01) and the similar distribution of their scores (Figure 1), makes it possible to compare the results of studies using the older version with studies using the revised version [8].

According to Bennet et al. [8], the FIQR has sound psychometric properties, discriminates between FMS patients, and patients with rheumatoid arthritis, systemic lupus erythematosus, or major depression disorder. Moreover, FIQR provides advantages over the previous version. The use of a numeric rating scale using 11 boxes, scored 0 to 10 in the FIQR as opposed to the combination of Likert and visual analogue scaling in the FIQ, considerably simplifies the scoring algorithm for the FIQR and obviates the need to use a ruler to measure visual analogue scales scores. This simplification and greater efficiency should make the FIQR easier to use by researchers and physicians. In our experience, the FIQR is substantially easier to use than the FIQ. The scoring system is much easier to perform and this facilitates its use not only by investigators but also in clinical grounds. Also, the substitution of the question “How have you felt when you get up in the morning?” by “Please rate the quality of your sleep” clarifies this question for the patients. In relation to the new added items, patients rated these symptoms in a similar range than the older ones; this means that they are present in most patients with fibromyalgia and, therefore, that they needed to be recorded, making this extension a welcome addition.

One of the limitations of our study is the fact that patients were recruited from fibromyalgia patients’ associations; this probably excluded less affected patients that frequently are not part of these associations. Also, our study did not assess some psychometric properties of the questionnaire, such as the sensitivity to change over time both with and without treatment. Finally, although patients were recruited in different parts of Spain in order ensure both a geographic and cultural diversity, it was not representative of every Spanish region.

## Conclusions

Despite the above mentioned limitations, we think that the Spanish FIQR can be currently considered a valid, usable and reliable tool for the assessment of Spanish-speaking FMS patients. It does not require prior specific training, and can be used both clinical and in research to be carried out on FMS patients.

## Abbreviations

FMS: Fibromyalgia syndrome; FIQ: Fibromyalgia Impact Questionnaire; FIQR: Revised Fibromyalgia Impact Questionnaire; CRF: Case Report Form; HADS: Hospital Anxiety and Depression Scale; SF-36: Short Form of 36-Health Survey; PCS: Physical component summary of SF-36; MCS: Mental component summary of SF-36; PF: Physical functioning; PR: Physical role functioning; BP: Bodily pain; GH: General health perceptions; VT: Vitality; SF: Social role functioning; RE: Emotional role functioning; MH: Mental health; BPI: Brief Pain Inventory; SD: Standard deviation; ICC: Intraclass correlation coefficient; CI: Confidence interval.

## Competing interests

The authors declare that they have no competing interests.

## Authors’ contributions

JMGL and EPC designed the project and performed the Spanish translation of the FIQR. MS, JMGL, JH, and RM contributed to the acquisition of data. JB performed the statistical analysis. MS and EPC interpreted the results and drafted the manuscript. All authors read and approved the final manuscript.

## Supplementary Material

Additional file 1 Spanish version of Revised Fibromyalgia Impact Questionnaire (FIQR). Click here for file
