# Supplementary material for: Validation of a Spanish version of the Revised Fibromyalgia Impact Questionnaire (FIQR)
Source: Health Qual Life Outcomes. 2013 Aug 1;11:132. doi: 10.1186/1477-7525-11-132 (PMC3770447; doi:10.1186/1477-7525-11-132)
Supplement: Additional file 1 — Spanish version of Revised Fibromyalgia Impact Questionnaire (FIQR). [file 1477-7525-11-132-S1.pdf]

Spanish version of Revised Fibromyalgia Impact Questionnaire (FIQR)

1.- Instrucciones: Para cada pregunta marque una **X** en la casilla que mejor **indique la dificultad con la que ha realizado cada una de las siguientes actividades a causa de la fibromialgia durante la última semana**

DIFICULTAD

a) Peinarse	**Ninguna** ☐ ☐ ☐ ☐ ☐ ☐ ☐ ☐ ☐ ☐ ☐ **Máxima**
b) Caminar 20 minutos sin necesidad de pararse	**Ninguna** ☐ ☐ ☐ ☐ ☐ ☐ ☐ ☐ ☐ ☐ ☐ **Máxima**
c) Preparar la comida	**Ninguna** ☐ ☐ ☐ ☐ ☐ ☐ ☐ ☐ ☐ ☐ ☐ **Máxima**
d) Barrer, fregar o pasar la aspiradora	**Ninguna** ☐ ☐ ☐ ☐ ☐ ☐ ☐ ☐ ☐ ☐ ☐ **Máxima**
e) Levantar y transportar una bolsa de la compra llena	**Ninguna** ☐ ☐ ☐ ☐ ☐ ☐ ☐ ☐ ☐ ☐ ☐ **Máxima**
f) Subir escaleras	**Ninguna** ☐ ☐ ☐ ☐ ☐ ☐ ☐ ☐ ☐ ☐ ☐ **Máxima**
g) Cambiar la ropa de la cama	**Ninguna** ☐ ☐ ☐ ☐ ☐ ☐ ☐ ☐ ☐ ☐ ☐ **Máxima**
h) Estar sentada en una silla durante 45 minutos	**Ninguna** ☐ ☐ ☐ ☐ ☐ ☐ ☐ ☐ ☐ ☐ ☐ **Máxima**
i) Hacer la compra	**Ninguna** ☐ ☐ ☐ ☐ ☐ ☐ ☐ ☐ ☐ ☐ ☐ **Máxima**

2.- Instrucciones: Para cada pregunta marque una **X** en la casilla que mejor **indique la influencia global que ha ejercido su fibromialgia en los últimos 7 días**

a) La fibromialgia me impidió hacer lo que tenía proyectado esta semana	**Nunca** ☐ ☐ ☐ ☐ ☐ ☐ ☐ ☐ ☐ ☐ ☐ **Siempre**
b) Los síntomas de mi fibromialgia me tuvieron totalmente abrumada	**Nunca** ☐ ☐ ☐ ☐ ☐ ☐ ☐ ☐ ☐ ☐ ☐ **Siempre**

3.- Instrucciones: Para cada pregunta marque una **X** en la casilla que mejor **indique la intensidad de los síntomas de su fibromialgia durante los últimos 7 días**

a) Dolor	**Ningún dolor** ☐ ☐ ☐ ☐ ☐ ☐ ☐ ☐ ☐ ☐ ☐ **Dolor insoportable**
b) Energía	**Muchísima energía** ☐ ☐ ☐ ☐ ☐ ☐ ☐ ☐ ☐ ☐ ☐ **Ninguna energía**
c) Rigidez	**Ninguna rigidez** ☐ ☐ ☐ ☐ ☐ ☐ ☐ ☐ ☐ ☐ ☐ **Rigidez intensa**
d) Calidad del sueño	**Me levanto descansada** ☐ ☐ ☐ ☐ ☐ ☐ ☐ ☐ ☐ ☐ ☐ **Me levanto muy cansada**
e) Depresión	**Nada deprimida** ☐ ☐ ☐ ☐ ☐ ☐ ☐ ☐ ☐ ☐ ☐ **Muy deprimida**
f) Problemas de memoria	**Buena memoria** ☐ ☐ ☐ ☐ ☐ ☐ ☐ ☐ ☐ ☐ ☐ **Muy mala memoria**
g) Ansiedad	**Nada ansiosa** ☐ ☐ ☐ ☐ ☐ ☐ ☐ ☐ ☐ ☐ ☐ **Muy ansiosa**
h) Dolorimiento al tacto	**Ninguno** ☐ ☐ ☐ ☐ ☐ ☐ ☐ ☐ ☐ ☐ ☐ **Muchísimo**
i) Problemas de equilibrio	**Ninguno** ☐ ☐ ☐ ☐ ☐ ☐ ☐ ☐ ☐ ☐ ☐ **Severos**
j) Grado de sensibilidad al ruido intenso, la luz brillante, los olores, el frío	**Ninguna sensibilidad** ☐ ☐ ☐ ☐ ☐ ☐ ☐ ☐ ☐ ☐ ☐ **Mucha sensibilidad**
